# Use of Antibiotic Treatment in Pregnancy and the Risk of Several Neonatal Outcomes: A Population-Based Study

**DOI:** 10.3390/ijerph182312621

**Published:** 2021-11-30

**Authors:** Anna Cantarutti, Federico Rea, Matteo Franchi, Benedetta Beccalli, Anna Locatelli, Giovanni Corrao

**Affiliations:** 1 National Centre for Healthcare Research and Pharmacoepidemiology, Department of Statistics and Quantitative Methods, University of Milano-Bicocca, 20126 Milan, Italy; federico.rea@unimib.it (F.R.); matteo.franchi@unimib.it (M.F.); giovanni.corrao@unimib.it (G.C.); 2 Unit of Biostatistics, Epidemiology and Public Health, Department of Statistics and Quantitative Methods, University of Milano-Bicocca, 20126 Milan, Italy; b.beccalli@campus.unimib.it; 3 Department of Mother and Child, ASST Vimercate, 20871 Vimercate, Italy; anna.locatelli@unimib.it; 4 School of Medicine and Surgery, University of Milano Bicocca, 20900 Monza, Italy

**Keywords:** antibiotic therapy, birth cohort, preterm birth, low birth weight, low Apgar score, pregnancy

## Abstract

Background: Limited evidence is available on the safety and efficacy of antimicrobials during pregnancy, with even less according to the trimester of their use. Objective: This study aimed to evaluate the association between exposure to antibiotics therapy (AT) during pregnancy and short-term neonatal outcomes. Methods: We considered 773,237 deliveries that occurred between 2007–2017 in the Lombardy region of Italy. We evaluated the risk of neonatal outcomes among infants that were born to mothers who underwent AT during pregnancy. The odds ratios and the hazard ratios, with the 95% confidence intervals, were estimated respectively for early (first/second trimester) and late (third trimester) exposure. The propensity score was used to account for potential confounders. We also performed subgroup analysis for the class of AT. Results: We identified 132,024 and 76,921 singletons that were exposed to AT during early and late pregnancy, respectively. Infants born to mothers with early exposure had 17, 11, and 16% increased risk of preterm birth, low birth weight, and low Apgar score, respectively. Infants that were exposed in late pregnancy had 25, 11, and 13% increased risk of preterm birth, low birth weight, and low Apgar score, respectively. The results were consistent in the subgroup analysis. Conclusion: Our results suggested an increased risk of several neonatal outcomes in women exposed to ATs during pregnancy, albeit we were not able to assess to what extent the observed effects were due to the infection itself. To reduce the risk of neonatal outcomes, women that are prescribed AT during pregnancy should be closely monitored.

## 1. Background

The most common infections that are encountered during pregnancy are those of the urinary tract and upper respiratory tract infections [1]. Untreated infections are associated with an increased risk of several neonatal outcomes, such as prematurity and low birth weight [2,3]. This explains why antibiotics are among the most used drugs during pregnancy, accounting for 39% of all dispensed medications during this period [4,5].

Several antibiotics are known to cross the placenta [6] and changes in antibiotics pharmacokinetics are expected during pregnancy [7]. Moreover, changes in the maternal microbiome resulting from antibiotic use may affect the maternal immune system [8] and convey modified bacterial flora to the fetus [9]. When taken together, these reasons explain the concerns about the implications of antibiotics use during pregnancy on adverse neonatal outcomes [10]. However, since pregnancy is often an exclusion criterion in clinical trials, and as few epidemiologic studies have been performed on this issue, limited evidence is available nowadays on the safety and efficacy of antimicrobials during pregnancy, with even less according to the trimester of their use.

We conducted a population-based cohort study in the Lombardy region of Italy to assess the potential association between the use of antibiotics during pregnancy and several short-term neonatal outcomes.

## 2. Methods

### 2.1. Setting

The data used for this study were retrieved from the healthcare utilization databases of Lombardy, which is a region of Italy that accounts for about 16% (almost ten million) of the national population. The Italian National Health Service (NHS) covers the entire population, and in Lombardy, it has been associated with an automated system of databases that were created to supply payments to providers of health services. These databases collect a variety of information including (i) demographic and administrative data of NHS beneficiary residents in Lombardy (approximately coinciding with the entire resident population); (ii) a database on hospital discharge records, including information about the primary diagnosis, co-existing conditions, and performed procedures (coded according to the ICD-9 CM classification system); (iii) an outpatient drug prescription database providing information on all community drugs that are reimbursed by the NHS (coded according to the Anatomical Therapeutic Chemical (ATC) classification system); (iv) a database reporting the Certificates of Delivery Assistance (i.e., the so-called CeDAP), which provide detailed information on the mother’s socioeconomic traits, as well as medical information on the pregnancy, childbirth, and child presentation at delivery. More details on the healthcare utilization databases that are used are reported elsewhere [11]. As a unique identification code is systematically used for all databases, their record linkage allows for providing a large and unselected birth cohort and establishing relevant traits and care pathways of mothers and newborns. To preserve privacy, each identification code was automatically de-identified, with the inverse process only being allowed by the Regional Health Authority on request from judicial authorities.

The specific diagnostic and therapeutic codes that were used for the current study are given in the Supplementary Materials (Table S1).

### 2.2. Study Cohort

**Figure 1:**
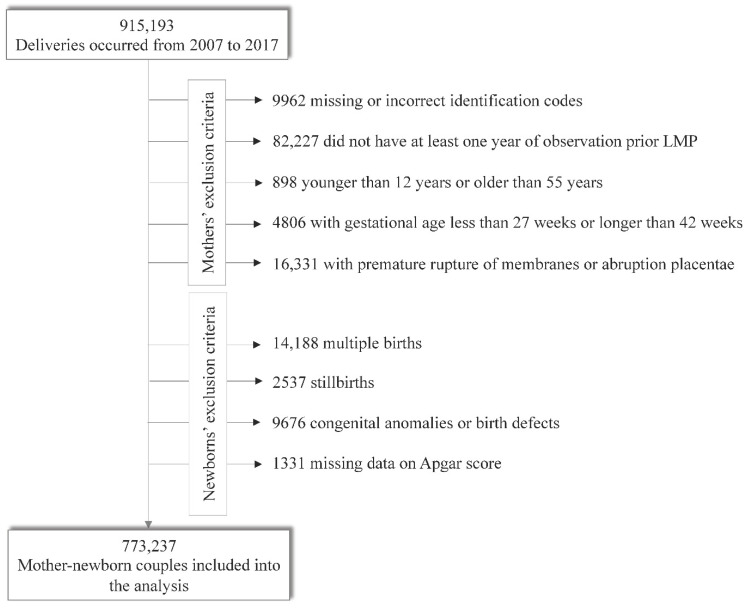
Flowchart of the inclusion and exclusion criteria.

The criteria for selecting the study cohort almost completely overlapped with those previously reported by our group [12,13,14]. Briefly, we considered singleton children born in Lombardy hospitals between 1 January 2007 and 31 December 2017, whose mother (i) was a beneficiary of the NHS and a resident in Lombardy for at least one year before the delivery, (ii) was aged 12 to 55 years at delivery, and (iii) had 27 to 42 weeks of gestation (gestational age was based on the last menstrual date). We excluded mothers who (i) did not have a hospital admission reporting an ICD-9-CM code for delivery, (ii) had multiple births, or (iii) experienced placenta abruption or premature rupture of membranes during the current pregnancy. We excluded pregnancies if the newborn (i) had a missing identification code, (ii) an Apgar score and/or birth weight was not reported on the CeDAP form, (iii) had chromosomal abnormalities, or (iv) was a stillbirth. Therefore, the final study population consisted of 773,237 mother–newborn couples (Figure 1).

### 2.3. Exposure to Antibiotics

Information on the antibiotics dispensed during pregnancy was obtained from the outpatient drug prescriptions database. Two mutually exclusive categories of antibiotic users were considered: (i) early users—if antibiotics were dispensed at least once during the first and/or second trimester of pregnancy, but no dispensations occurred during the third trimester of pregnancy; and (ii) late users—if antibiotics were dispensed at least once during the third trimester, regardless of the use during the first/second trimesters. Women who never used antibiotics, and those who did not use antibiotics during the third trimester of pregnancy, were considered as reference categories (i.e., unexposed) of early and late users, respectively (Figure S1 in the Supplementary Materials).

### 2.4. Outcomes

Neonatal outcomes that were diagnosed at presentation were identified from the CeDAP database. The outcomes of interest were preterm birth (<37 gestation weeks) [15], low birth weight (<2500 g) [16], small for gestational age [17], and low 5 min Apgar score (<7) [18] (Table S2 in the Supplementary Materials).

### 2.5. Maternal Covariates

Maternal traits, including sociodemographic features (i.e., age at delivery, nationality, marital status, education, employment, previous miscarriages, and parity) and gestational age, were obtained from the CeDAP database. Selected medical morbidities (i.e., diabetes; hypertension; preeclampsia; dyslipidemia; neuropathic, non-neuropathic, and other pain; obesity or overweight; substance dependence; and infection), concomitant medications (i.e., non-steroidal anti-inflammatory drugs and drugs for acid-related disorders), and use of healthcare services (number of hospitalizations and distinct prescription drugs, excluding antibiotics) were identified. The sources were the CeDAP, hospital discharge, and outpatient drug prescription databases where appropriate.

### 2.6. Statistical Analyses

Early and late users of antibiotics were compared with no users for all the covariates listed above through the standardized mean difference (SMD), which is an effect size measurement. This measure is defined as the mean difference divided by the pooled standard deviation [19]. In contrast to statistical hypothesis tests (e.g., *t*-tests and chi-square tests), the SMD is not influenced by the sample size. An SMD > 0.1 denotes an imbalance between groups.

A logistic regression model was fitted for estimating the odds ratio (OR) and the corresponding 95% confidence interval (CI) of the association between the early use of antibiotics and each of the considered neonatal outcomes. To avoid immortal time bias [20,21], the late use–outcome association was estimated through a Cox proportional hazard model in which late antibiotics exposure was considered as a time-varying covariate.

We used the propensity score (PS) in the attempt of between-group balancing. We calculated the PS, namely, the predicted probability of antibiotics dispensing, separately for early and late users through logistic regression models that included all the aforementioned covariates. Two PSs were generated: (i) partially adjusted PS that included all maternal characteristics except socio-demographic features, and (ii) a fully adjusted PS that also included socio-demographic features. The PS so calculated was included as a covariate into both logistic and Cox models. Because socio-demographic data was missing from some CeDAP forms (on average, this occurred in 0.9 to 3.9% of them), only records reporting complete information were used for estimating the fully adjusted PS.

### 2.7. Sensitivity and Subgroup Analyses

Subgroup and sensitivity analyses were performed to evaluate the robustness of the main analysis. First, an excess risk of the considered neonatal outcomes was reported in women that had a cesarean delivery [22,23,24] and since cesarean delivery may be on the causal path between antibiotic exposure and the neonatal events, we conducted subgroup analyses that were restricted to vaginal deliveries. Second, we performed a subgroup analysis for specific classes of antibiotics [25]. Between-strata homogeneity of ORs and HRs was tested [26].

## 3. Results

### 3.1. Study Cohort and Outcomes

Out of 773,237 women included in the study cohort, 208,945 (27%) received antibiotics during pregnancy, of whom, 132,024 (63%) were classified as early users, and the remaining 76,921 (37%) were late users (Table S3 in the Supplementary Materials).

With the exception of a higher prevalence of antibiotic users among women with low education, who were unemployed, who had a C-section, and who concomitantly used other drugs, there was no substantial difference between both early and late users of antibiotics and the corresponding no users (Table 1).

Among the 773,237 pregnancies, 31,061 (4%) had a preterm birth, 33,953 (4.4%) had a low birth weight, 56,168 (7.3%) was small for gestational age, and 3472 (0.4%) had a low Apgar score (Table S3 in the Supplementary Materials).

### 3.2. Use of Antibiotics and Neonatal Outcomes

**Figure 2:**
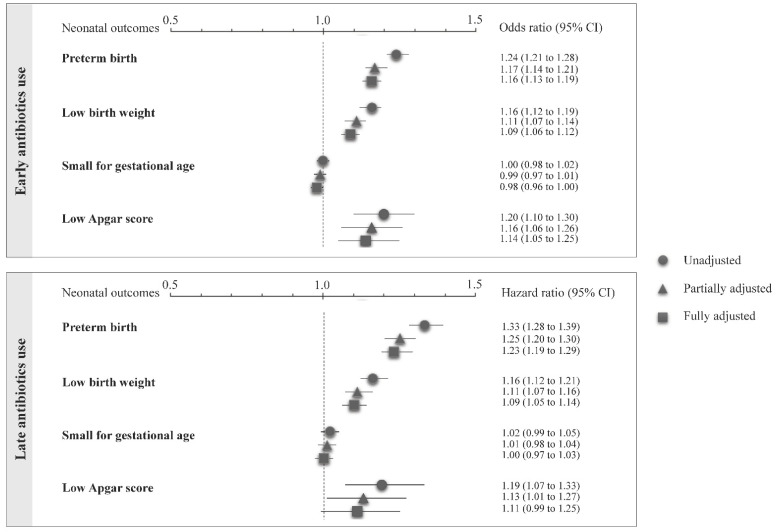
Odds and hazard ratios (and their 95% confidence intervals) of selected outcomes associated with early and late use of antibiotics during pregnancy, respectively.

With the exception of small for gestational age, all other neonatal outcomes were significantly associated with both early and late use of antibiotics (Figure 2). The odds ratios and hazard ratios did not differ substantially according to unadjusted, partially adjusted, or fully adjusted estimates. The fully adjusted estimates showed risk excesses ranging from 9% (95% CI: 6–12%) for low birth weight to 16% (13–19%) for preterm birth in early exposed women. While the risk excesses in late-exposed women range from 9% (5–14%) for low birth weight to 23% (19–29%) for preterm birth. The fully adjusted estimate of the association between late use and low Apgar did not reach the conventional level of statistical significance, which was likely because of the low power of the investigated association.

Odds ratios and their 95% confidence intervals were estimated with logistic regression, including early exposure to antibiotics as a time-fixed covariate. Hazard ratios and their 95% confidence intervals were estimated with a Cox proportional hazard model, including late exposure to antibiotics as a time-varying covariate. Unadjusted, partially adjusted, and fully adjusted estimates are reported (see text for further details).

**Figure 3:**
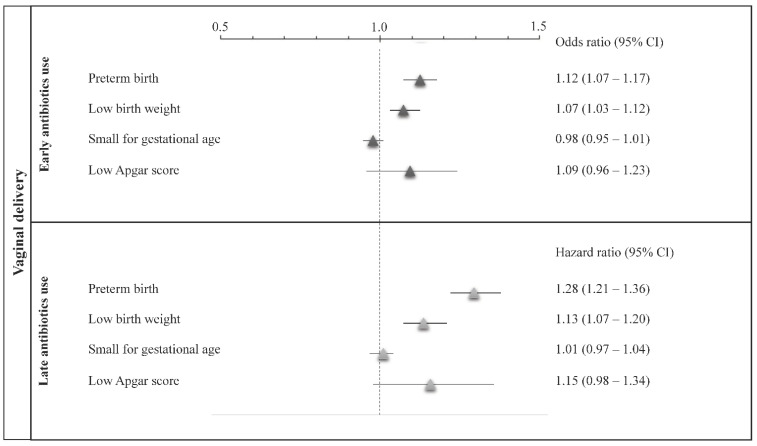
Odds and hazard ratios (and their 95% confidence intervals) of selected outcomes associated with early and late use of antibiotics during pregnancy, respectively, among women who had a vaginal delivery.

Similar findings were observed by restricting the cohort to those women who had a vaginal delivery (Figure 3), although only preterm birth and low birth weight, but not small for gestational age and a low 5 min Apgar score, were significantly associated with both the early and late use of antibiotics. Finally, there was evidence that both the early and late use of specific classes of antibiotics differently affected the risk of preterm birth and low birth weight (Table 2). In particular, women who used macrolides and cephalosporins during the third trimester of pregnancy had 58% (44–73%) and 40% (22–61%) increased risks of preterm birth relative to those who did not use macrolides and cephalosporins during this period, respectively. In addition, women who used cephalosporins early had 23% (12–36%) and 10% (1–22%) increased risks of preterm birth and low birth weight, respectively, than those who never used cephalosporins during pregnancy. There was no evidence that the use of fluoroquinolones affected the risk of either preterm birth or low birth weight.

Odds ratios and their 95% confidence intervals were estimated with logistic regression, including early exposure to antibiotics as a time-fixed covariate. Hazard ratios and their 95% confidence intervals were estimated with a Cox proportional hazard model, including late exposure to antibiotics as a time-varying covariate. Unadjusted, partially adjusted, and fully adjusted estimates are reported (see text for further details).

## 4. Discussion

Our large population-based study offered evidence that women who were prescribed antibiotics during pregnancy were at higher risk of preterm birth, and some of its correlates, mainly low birth weight and perhaps a 5 min low Apgar score. These effects were not negligible since, compared to non-users, women who used antibiotics had an excess risk of 16% and 23% (preterm birth associated with early and late use, respectively), 9% (low birth weight associated with both early and late use), and 13% (low 5 min Apgar score associated with early use). Our findings confirmed and extended the results of prior studies. In terms of the effect of antibiotic exposure during pregnancy, our results were consistent with those from Vidal et al. who demonstrated a lower birth weight in infants who were born to women who used antibiotic therapies during pregnancy compared to unexposed infants, and with those from Mensah et al. who found a lower mean Apgar score among infants who were exposed to antibiotic during the 24 h before delivery [27,28]. In contrast, Jepsen et al. did not find any increased risk of adverse pregnancy outcomes in infants who were exposed to amoxicillin during pregnancy compared with unexposed infants [29].

According to the available evidence, penicillins and cephalosporins are considered safe to use in pregnancy (these antibiotics are the first-line options for many infections in pregnancy), while the use of fluoroquinolones should be avoided unless the benefits outweigh the risks [25,30,31]. More uncertainty remains for the use of macrolides during pregnancy [25,32]. We observed heterogeneous class effects of antibiotics, but with safety profiles that were inconsistent with the available evidence. Indeed, the action of fluoroquinolones, penicillins, macrolides, and cephalosporins on preterm birth and low birth weight was negligible for the first class and progressively increased for the following classes. More studies are needed to confirm these trends.

Although there is a consensus on the role of intra-amniotic infection as the main cause of preterm births [33,34,35,36,37,38], currently, there is no evidence supporting antibiotic prophylaxis for reducing the risk of preterm delivery and other maternal and newborn adverse outcomes due to infection [39,40]. Rather, as antibiotics are prescribed mostly as therapy, their use can be thought of as a proxy of the onset of some kind of infection. Accordingly, the more likely explanation of our results is that the observed excess of adverse neonatal outcomes among exposed women may be due to the indirect action of the infection (which induced the antibiotic use) rather than a direct effect of the drug. However, since some of them are known to cross the placenta [6], we cannot exclude the possibility that antibiotics directly acted on adverse neonatal outcomes. On the other hand, the observed heterogeneous class effect did not help to discriminate between the indirect and direct action of the use of antibiotics in pregnancy on adverse neonatal outcomes. The latter could, in fact, be due to differences in (i) indication for antibiotic treatment, (ii) ability to cross the placenta, and (iii) action in inhibiting the intestinal flora. Anyhow, our results suggested careful clinical attention for women who developed an infection during pregnancy, primarily in the clinical decision regarding whether to administer antibiotics and on the type of antibiotic to dispense. Indeed, although it is known that untreated infection during pregnancy is associated with the risk of several neonatal outcomes, such as prematurity and low birth weight [2,3], our findings suggest that the use of antibiotics was not sufficient to nullify the risk of neonatal outcomes in infants who were exposed to infection during pregnancy.

The current study had several strengths. First, this was a very large population-based cohort study, which provided the statistical power to evaluate the effect of the timing of antibiotic use on several neonatal outcomes. Second, our conclusions were based on converging evidence from several models that accounted for several measured confounding factors, including maternal socio-demographic characters. Finally, the robustness of the findings was assessed using several sensitivity and subgroup analyses.

The study limitations included the potential selection bias, misclassification of the exposure, and residual confounding.

First, since we excluded mothers that were too young and too old, those with premature rupture of membranes and abruption of the placenta, pregnancies with less than 27 gestational weeks, and childbirths resulting in multiple births and stillbirths, the generalizability of our findings may be questionable since they involved healthier mother–child pairs. Second, our study was based on the assumption that a filled prescription implied utilization of that treatment, which is not always true. Third, women that used a C-section delivery should always be subjected to antibiotic therapy before or after cord clamping and no studies have investigated the short-, mid-, and long-term effects on infants [41]; unfortunately, we were not able to deal with this issue since we did not record the antibiotics dispensed during hospitalization, resulting in a loss of information and misclassification of exposure. Nevertheless, restricting the analysis to vaginal deliveries confirmed our results, suggesting that the type of delivery did not have the role of mediator in the antibiotics → neonatal outcomes relationship.

Fourth, due to the observational design of our study, it could not fully rule out all confounders. For example, lifestyle factors, such as alcohol abuse and smoking, as well as unmeasured socio-demographic features, are known to be under-recorded in administrative databases, in addition to some drugs (e.g., steroids) that are handed out over the counter or by hospitals and, thus, they were not retrievable by our databases [42].

Finally, our data did not allow us to highlight the mechanism underlying the observed associations, i.e., to quantify the extent to which the observed associations between the use of antibiotics and neonatal outcomes were explained by the infection (which caused the use of the drug).

What is already known about this subject:

- Limited evidence is available on the safety and efficacy of antimicrobials during pregnancy.
- The concerns about the implications of antibiotics use during pregnancy on adverse neonatal outcomes involve the need for observational studies to assess the potential association between the use of antibiotics during pregnancy and several neonatal outcomes.

What this study adds:

- Our results suggested an increased risk of several neonatal outcomes in women who were exposed to antibiotics during pregnancy.
- How the observed effects are due to the infections or the direct action of the considered antimicrobial drugs remains an important open question.

## 5. Conclusions

In conclusion, our results suggested that women who used antibiotics during pregnancy, mainly macrolides, penicillins, and cephalosporins, were at higher risk of preterm birth, low birth weight, and perhaps a low Apgar score. We could not assess to what extent the observed effects were due to the infection that caused the antibiotics dispensation, the direct action of the considered antimicrobial drugs, or both of these reasons. However, as antibiotics are among the most used drugs during pregnancy, efforts that are aimed at elucidating the risk–benefit profiles in this field have major implications for public health and are therefore urgent and important tasks. In the meantime, women who develop an infection and use antibiotics during pregnancy should receive careful clinical attention to reduce the risk of adverse neonatal outcomes.

## Figures and Tables

**Table 1:** Comparison of selected maternal characters among early and late users of antibiotics relative to the corresponding reference.

Maternal Characteristics	Early Users ^(a)^	No Users ^(a)^	SD	Late Users ^(b)^	No Users ^(b)^	SD
	(N = 132,024)	(N = 564,292)		(N = 76,921)	(N = 696,316)	
Socio-demographics						
Age, mean (SD) ^(c)^	32.7 (5.3)	32.3 (5.1)	0.08	32.2 (5.4)	32.4 (5.1)	−0.04
No Italian nationality ^(c,d)^	29,180 (23.03%)	121,975 (22.48%)	0.01	18,725 (25.39%)	151,155 (22.58%)	0.07
Unmarried status ^(c,d)^	40,353 (31.85%)	167,681 (30.9%)	0.02	22,615 (30.66%)	208,034 (31.08%)	−0.01
Education lower than high school ^(c,d)^	35,523 (28.03%)	133,125 (24.53%)	0.08	22,842 (30.97%)	168,648 (25.2%)	0.13
Unemployed ^(c,d)^	35,892 (28.32%)	142,578 (26.27%)	0.05	23,772 (32.23%)	178,470 (26.66%)	0.12
Previous miscarriages ^(c)^	34,601 (26.21%)	134,620 (28.86%)	−0.06	20,505 (26.66%)	169,221 (24.3%)	0.05
Primigravida ^(c,d)^	37,068 (29.25%)	17,4241 (32.11%)	−0.06	20,601 (27.93%)	211,309 (31.57%)	−0.08
Comorbidities ^(e)^						
Substance dependence	52 (0.04%)	155 (0.03%)	0.01	37 (0.05%)	207 (0.03%)	0.01
Infection	4383 (3.32%)	11,955 (2.12%)	0.07	4674 (6.08%)	27,942 (4.01%)	0.09
Hypertension	232 (0.18%)	842 (0.15%)	0.01	118 (0.15%)	1074 (0.15%)	0
Preeclampsia	105 (0.08%)	393 (0.07%)	0	61 (0.08%)	498 (0.07%)	0
Diabetes	328 (0.25%)	1325 (0.22%)	0.01	211 (0.27%)	1563 (0.22%)	0.01
Obesity and overweight	149 (0.11%)	372 (0.07%)	0.01	123 (0.16%)	521 (0.07%)	0.03
Dyslipidaemia	12 (0.01%)	37 (0.01%)	0	10 (0.01%)	49 (0.01%)	0
Pain	609 (0.46%)	2135 (0.38%)	0.01	399 (0.52%)	2744 (0.39%)	0.02
C-section ^(f)^	39,092 (29.61%)	150,491 (26.67%)	0.07	21,449 (27.88%)	189,583 (27.23%)	0.01
Concomitant medications ^(e)^						
NSAIDs	8680 (6.57%)	23,117 (4.1%)	0.11	5171 (6.72%)	31,797 (4.57%)	0.09
Drugs for acid-related disorders	14,264 (10.8%)	38,733 (6.86%)	0.14	8274 (10.73%)	52,997 (7.61%)	0.11
Healthcare utilization ^(e)^						
Hospital admissions	26,706 (20.23%)	106,000 (18.78%)	0.04	1586 (20.62%)	132,706 (19.06%)	0.04
No. of distinct prescription drugs, excluding antibiotics						
=1	40,903 (30.98%)	16,8528 (29.87%)	0.02	23,531 (30.59%)	209,431 (30.08%)	0.01
≥2	43,965 (33.3%)	12,6129 (22.35%)	0.25	24,831 (32.28%)	170,094 (24.43%)	0.17

SD: standardized difference; ^(a)^ early users (women whom antibiotics were dispensed at least one antibiotic during the first and/or second trimester of pregnancy but not during the thirst trimester) and the corresponding referent (women who never used antibiotics during pregnancy); ^(b)^ late users (women to whom antibiotics were dispensed at least once during the third trimester, regardless of the use during the first/second trimesters) and the corresponding referent (women who did not use antibiotics during the third trimester of pregnancy); ^(c)^ at the date of the current delivery; ^(d)^ because some data was missing, socio-demographic features refer to 113,757 early users (and the corresponding 485,766 referents) and 73,089 late users (and the corresponding 616,858 referents); ^(e)^ from one year before the last menstrual date through to the end of the first trimester of pregnancy; ^(f)^ current pregnancy.

**Table 2:** Effect of the early and late use of specific classes of antibiotics on the risks of preterm birth, low birth weight, small for gestational age, and low 5 min Apgar score.

	Early Users			Late Users		
	Entire Cohort	Women Who Experienced Neonatal Outcome	OR (95% CI)	Entire Cohort	Women Who Experienced Neonatal Outcome	HR (95% CI)
Preterm birth						
Cephalosporins	7816 (1.1%)	429 (5.5%)	1.23 (1.12–1.36)	4113 (0.5%)	204 (4.9%)	1.40 (1.22 to 1.61)
Penicillins	84,211 (12.1%)	3970 (4.7%)	1.12 (1.08–1.16)	45,285 (5.9%)	1679 (3.7%)	1.12 (1.07 to 1.18)
Macrolides	24,763 (3.6%)	1303 (5.3%)	1.22 (1.15–1.30)	8402 (1.1%)	472 (5.6%)	1.58 (1.44 to 1.73)
Fluoroquinolones	6352 (0.9%)	310 (4.9%)	1.08 (0.96–1.21)	1340 (0.2%)	57 (4.2%)	1.21 (0.93 to 1.57)
Other	24,715 (3.6%)	1243 (5.0%)	1.18 (1.11–1.25)	12,158 (1.6%)	510 (4.2%)	1.23 (1.13 to 1.53)
Homogeneity test (*p*-value)			0.0448			<0.0001
Low birth weight						
Cephalosporins	7816 (1.1%)	411 (5.3%)	1.10 (1.00–1.22)	4675 (0.6%)	217 (4.6%)	1.26 (1.11 to 1.44)
Penicillins	84,211 (12.1%)	4050 (4.8%)	1.05 (1.01–1.08)	52,923 (6.8%)	1967 (3.7%)	1.05 (0.99 to 1.09)
Macrolides	24,763 (3.6%)	1347 (5.4%)	1.17 (1.11–1.24)	9316 (1.2%)	462 (5.0%)	1.33 (1.21 to 1.46)
Fluoroquinolones	6352 (0.9%)	331 (5.2%)	1.09 (0.97–1.21)	1550 (2.2%)	71 (4.6%)	1.25 (0.99 to 1.58)
Other	24,715 (3.6%)	1317 (5.3%)	1.16 (1.09–1.23)	13,885 (1.8%)	534 (3.8%)	1.05 (0.96 to 1.14)
Homogeneity test (*p*-value)			0.0026			<0.0001
Small for gestational age						
Cephalosporins	7816 (1.1%)	575 (7.4%)	0.99 (0.92 to 1.09)	4675 (0.6%)	345 (7.4%)	1.10 (0.99 to 1.22)
Penicillins	84,211 (12.1%)	6045 (7.2%)	0.97 (0.94 to 1.00)	52,923 (6.8%)	3696 (7.0%)	1.00 (0.96 to 1.03)
Macrolides	24,763 (3.6%)	1858 (7.5%)	1.02 (0.97 to 1.07)	9316 (1.2%)	673 (7.2%)	1.07 (0.99 to 1.15)
Fluoroquinolones	6352 (0.9%)	466 (7.3%)	0.99 (0.90 to 1.09)	1550 (2.2%)	126 (8.1%)	1.15 (0.97 to 1.37)
Other	24,715 (3.6%)	1864 (7.5%)	1.03 (0.98 to 1.09)	13,885 (1.8%)	960 (6.9%)	0.98 (0.92 to 1.05)
Homogeneity test (*p*-value)			0.2032			0.0971
Low 5 min Apgar score						
Cephalosporins	7816 (1.1%)	50 (0.6%)	1.33 (1.00 to 1.76)	4675 (0.6%)	16 (3.7%)	0.84 (0.51 to 1.38)
Penicillins	84,211 (12.1%)	417 (0.5%)	1.07 (0.96 to 1.18)	52,923 (6.8%)	234 (0.4%)	1.10 (0.96 to 1.26)
Macrolides	24,763 (3.6%)	123 (0.5%)	1.05 (0.88 to 1.26)	9316 (1.2%)	50 (0.5%	1.35 (1.02 to 1.79)
Fluoroquinolones	6352 (0.9%)	36 (0.6%)	1.16 (0.83 to 1.61)	1550 (2.2%)	3 (0.2%)	0.45 (0.14 to 1.40)
Other	24,715 (3.6%)	151 (0.6%)	1.32 (1.12 to 1.56)	13,885 (1.8%)	70 (0.5%)	1.24 (0.97 to 1.57)
Homogeneity test (*p*-value)			0.1782			0.1961

## Data Availability

The data that support the findings of this study are available from the Lombardy Region, but restrictions apply to the availability of these data, which were used under license for the current study and, thus, are not publicly available. Data are however available from the Lombardy Region upon reasonable request.

## Supplementary Materials

The following are available online at https://www.mdpi.com/article/10.3390/ijerph182312621/s1, Figure S1: Exposure definition. Table S1: Specific diagnostic and therapeutic codes used for the current study; Table S2: Distribution of neonatal outcomes; Table S3: Distribution of the Early and Late exposure.
