# Supplementary material for: Use of Antibiotic Treatment in Pregnancy and the Risk of Several Neonatal Outcomes: A Population-Based Study
Source: Int J Environ Res Public Health. 2021 Nov 30;18(23):12621. doi: 10.3390/ijerph182312621 (PMC8657211; doi:10.3390/ijerph182312621)
Supplement: Supplementary file 1 [file ijerph-18-12621-s001.zip › ijerph-1461315-supplementary.pdf]

Supplementary material

This appendix has been provided by the authors to give readers additional information about their work.

Table S1 Specific diagnostic and therapeutic codes used for the current study

- ICD-9 Diagnostic codes for delivery

**ICD-9 codes**	
ICD-9 Diagnostic codes	v27.xx or 640.xy – 676.xy (where y=1 or 2)
ICD-9 Procedures codes	72.x, 73.2, 73.5, 73.6, 73.8, 73.9, 74.0, 74.1, 74.2, 74.4, 74.99

- ICD-9 Diagnostic codes for premature rupture of membranes and placenta abruption

**ICD-9 codes**	
Premature rupture of membranes	658.1x, 658.2x, 761.1
Placenta abruption	641.2x, 762.1

- ICD-9 Diagnostic codes for congenital malformation

**ICD-9 codes**	
Congenital malformation	740 - 759

- ICD-9 Diagnostic codes for miscarriage and/or stillbirth

**ICD-9 codes**	
Miscarriage	634-639
Stillbrith	656.4, V27.1, V27.4, V27.7

- ATC codes for the prescription of antibiotics

**ATC CODE**	**Antibiotic classes**
J01A	Tetracyclines
J01B	Amphenicols
J01C	Beta-lactam antibacterials, penicillins
J01D	Other beta-lactam antibacterials
J01E	Sulfonamides and trimethoprim
J01F	Macrolides, lincosamides and streptogramins
J01G	Aminoglycoside antibacterials
J01M	Quinolone antibacterials
J01R	Combinations of antibacterials
J01X	Other antibacterials

Figure S1 Exposure definition

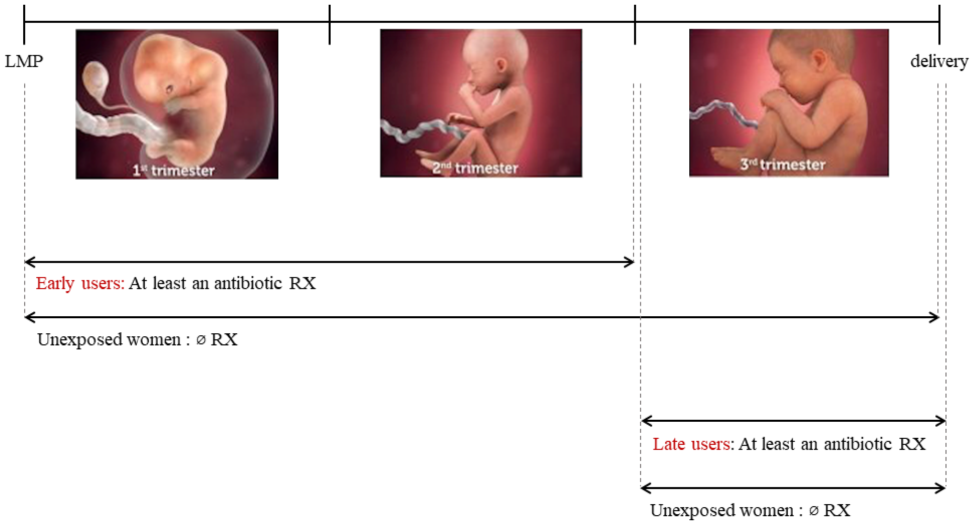

Table S2. Distribution of neonatal outcomes

**PTB**	**LBW**	**SGA**	**Low Apgar**	No. Of women	(%)
				685,981	(88.72)
				2365	(0.31)
				35,340	(4.57)
				183	(0.02)
				1398	(0.18)
				4	(0.00)
				16,771	(2.17)
				134	(0.02)
				15,268	(1.97)
				147	(0.02)
				11,406	(1.48)
				500	(0.06)
				3601	(0.47)
				139	(0.02)

Table S3. Distribution of the Early and Late exposure

Early exposure	Late exposure	No. Of women	(%)
		564,292	(72.98)
		49,959	(6.46)
		132,024	(17.07)
		26,962	(3.49)
